# Supplementary material for: Comparison of thyroid hormones reference intervals based on thyroid antibody levels: A multicenter study
Source: PLoS One. 2026 Mar 18;21(3):e0344197. doi: 10.1371/journal.pone.0344197 (PMC12998823; doi:10.1371/journal.pone.0344197)
Supplement: Attachment — Submitted filename: Response to Reviewers- plosone 19.01.2026.docx [file pone.0344197.s004.docx]

**Comments to the Author**

1. Is the manuscript technically sound, and do the data support the conclusions?

The manuscript must describe a technically sound piece of scientific research with data that supports the conclusions. Experiments must have been conducted rigorously, with appropriate controls, replication, and sample sizes. The conclusions must be drawn appropriately based on the data presented.

Reviewer #1: Partly

2. Has the statistical analysis been performed appropriately and rigorously?

Reviewer #1: Yes

3. Have the authors made all data underlying the findings in their manuscript fully available?

The [PLOS Data policy](http://track.editorialmanager.com/CL0/http:%2F%2Fwww.plosone.org%2Fstatic%2Fpolicies.action%23sharing/1/010f019b3563d73b-b5edb0a9-171e-4ce6-a4fd-380ca549c77e-000000/NrMwSuq0S7yT-erS_5eTzYjiVROr2kqURleGp2oN4lA=242) requires authors to make all data underlying the findings described in their manuscript fully available without restriction, with rare exception (please refer to the Data Availability Statement in the manuscript PDF file). The data should be provided as part of the manuscript or its supporting information, or deposited to a public repository. For example, in addition to summary statistics, the data points behind means, medians and variance measures should be available. If there are restrictions on publicly sharing data—e.g. participant privacy or use of data from a third party—those must be specified.

Reviewer #1: No

4. Is the manuscript presented in an intelligible fashion and written in standard English?

PLOS ONE does not copyedit accepted manuscripts, so the language in submitted articles must be clear, correct, and unambiguous. Any typographical or grammatical errors should be corrected at revision, so please note any specific errors here.

Reviewer #1: Yes

5. Review Comments to the Author

Please use the space provided to explain your answers to the questions above. You may also include additional comments for the author, including concerns about dual publication, research ethics, or publication ethics. (Please upload your review as an attachment if it exceeds 20,000 characters)

Reviewer #1: The manuscript entitled “Comparison of Thyroid Hormones Reference Intervals Based on Thyroid Antibody Levels: A multicenter study” aims to determine reference intervals for thyroid hormones based on thyroid antibody levels using different autoanalyzer kits.

This study addresses the fact of being one of the few multicenter studies to establish antibody specific RI for thyroid hormones across four different autoanalyzers.

Regarding the novelty of the article, there are several publications that have previously established RIs for thyroid hormones using the refineR algorithm, some of them have included very large datasets and multiple analyzers as well:

- M.K. Bohn, D. Bailey, C. Balion, G. Cembrowski, C. Collier, V. De Guire, V. Higgins, B. Jung, Z.M. Ali, D. Seccombe, J. Taher, A.K.Y. Tsui, A. Venner, K. Adeli, Reference interval harmonization: harnessing the power of big data analytics to derive common reference intervals across populations and testing platforms, Clin Chem 69 (9) (2023) 991–1008.

-Lewis CW, Raizman JE, Higgins V, Gifford JL, Symonds C, Kline G, Romney J, Doulla M, Huang C, Venner AA. Multidisciplinary approach to redefining thyroid hormone reference intervals with big data analysis. Clin Biochem. 2024 Dec;133-134:110835.

-Helen I.Jansen et al. Age-Specific Reference Intervals for Thyroid-Stimulating Hormones and Free Thyroxine to Optimize Diagnosis of Thyroid Disease. doi: 10.1089/thy.2024.0346.

In regards of the design of the study, it is unclear if thyroid diseases have been properly excluded and could affect the thyroid antibody positive subgroup since the exclusion criteria were based solely on medication treatment and not on thyroid disease. It is recommended to exclude positive thyroid disease patients as exclusion criteria instead of only performing it based on medication treatment.

**Response:** Our exclusion methodology was comprehensive and included both clinical diagnoses and medication use. Specifically, patients with documented thyroid disease diagnoses (based on hospital Laboratory Information System and Hospital Information System records) and/or those receiving thyroid-related medications were systematically excluded from the study. This dual-approach methodology ensures that thyroid disease status was captured regardless of whether patients had formal diagnostic documentation or were being treated with thyroid medications at the time of enrollment. The manuscript text has been revised to clearly articulate this exclusion strategy and to emphasize that thyroid disease screening was not limited solely to medication-based criteria.

If available to the researchers, another exclusion criteria that could be considered would be abnormal thyroid ultrasound results.

**Response:** In our study, patients for whom thyroid ultrasonography was requested in the Hospital Information System and who were subsequently diagnosed with thyroid disease based on imaging findings were excluded from the analysis.

In regards of thyroid antibodies, were positive anti-thyroid receptor antibodies considered a motive of exclusion?

**Response:** Thyroid receptor antibody (TRAb) positivity alone was not used as an exclusion criterion in our study. However, patients with a documented diagnosis of Graves' disease, which is characterized by TRAb positivity and associated clinical findings, were systematically excluded from the study.

In regards of exclusion criteria based on medication treatment, did you consider the radioactive iodine (I-131) treatment?

**Response:** We confirm that our exclusion criteria comprehensively addressed all thyroid-related treatments. Patients who had received any thyroid-related medication or treatment modalities, including radioactive iodine (I-131) therapy, were excluded from the study.

Did you only include primary care patients, or did you include hospitalized patients?

**Response:** Patients from intensive care units and emergency departments, as well as those whose samples were collected in the afternoon or evening due to the circadian rhythm of TSH, were excluded from the study.

Our study included only primary care outpatients to ensure a representative community-based population. Patients from intensive care units and emergency departments were excluded due to the acute nature of their conditions and potential physiological stress affecting thyroid hormone levels. Additionally, samples collected in the afternoon or evening were excluded from the analysis to minimize the effect of circadian rhythm variations on TSH measurements, as TSH levels demonstrate significant diurnal variation.

Were results from a single patient excluded from the analysis when multiple results were obtained for that patient within the same year (repeated measurements) to reduce the percentage of pathological results in the datasets?

**Response:** Yes, we carefully addressed the issue of repeated measurements. When multiple samples from the same patient were present in the system within the same year, only the first non-pathological sample was included in the analysis. This approach prevents the overrepresentation of pathological results and ensures that each patient contributes only one measurement to the dataset, thereby maintaining the statistical integrity and reducing potential bias from repeated testing.

Did you take into consideration if reflex-testing for assessment of thyroid function could affect your results? A number of laboratories use reflex-testing for assessment of thyroid function, triggering FT4 automatically when TSH results are outside the RI.

**Response:** In our country, reflex testing protocols for automatic FT4 measurement when TSH is outside the reference interval are not implemented due to national laboratory regulations and guidelines. Nevertheless, we recognize that in laboratory settings where reflex testing is routinely used, this could influence thyroid function test patterns. This consideration has been added to the limitations section of the manuscript.

I would recommend excluding results from specialists, inpatients, or repeat testing.

**Response:** We appreciate this recommendation and confirm that our exclusion criteria align with this suggestion. Data from inpatients, including those from intensive care units, endocrinology, nephrology, and emergency departments, were systematically excluded from the study to ensure a representative primary care population. Additionally, as previously detailed, when repeat testing occurred within the same year for individual patients, only the first non-pathological result was retained for analysis. These stringent exclusion criteria were implemented to minimize potential confounding factors and ensure the study population reflects routine primary care laboratory testing patterns rather than specialist or acute care settings. The manuscript has been revised to clearly articulate these exclusion strategies.

Could you provide the pathological fraction from the refineR statistics for each platform?
**Response:** Yes, as requested, the pathological fractions obtained from the refineR statistics for each platform have been comprehensively added as a supplementary table in the Supplementary Material section.

Being a study performed during several years, please state if significant drift in test result measurement due to reagent reformulation, population drift, seasonal variation, and/or reagent or calibrator lot-to-lot variation/reformulation was taken into consideration.

**Response:** We acknowledge this is an important methodological consideration for longitudinal studies. Throughout the study period, the same analytical analyzers were consistently used across all systems to maintain standardization. However, we recognize that different reagent lots were applied during the study duration, which may have influenced individual test results.

We believe that accounting for lot-to-lot variation and seasonal variability actually contributes to obtaining more realistic and clinically applicable reference intervals, as these variations reflect real-world laboratory practice. Nevertheless, we acknowledge that reagent reformulations and lot-to-lot variations represent potential sources of systematic variation that could affect reference interval establishment. The potential effects of reagent lot changes, calibrator variations, and seasonal fluctuations have been explicitly added to the limitations section of the manuscript to ensure transparency regarding these confounding factors.

Please mention if the internal quality control data set was used to ensure the correctness and reliability of the results, and if the laboratories are accredited by the international organization for standardization 15,189 (ISO 15189).

**Response:** One of our hospitals, a private institution, is ISO 15189 accredited. Nevertheless, similar quality requirements (including both internal quality control – IQC and external quality assessment – EQA programs) are mandatory in Turkey and are regularly monitored. During the study period, all laboratories consistently performed the necessary quality control procedures.

We appreciate this important question regarding quality assurance. One of our participating hospitals, a private institution, is ISO 15189 accredited, demonstrating compliance with international standards for medical laboratory competence.

For all participating laboratories, whether ISO 15189 accredited or not, similar rigorous quality requirements are mandatory in Turkey and are regularly monitored by national regulatory authorities. These include both internal quality control (IQC) procedures and external quality assessment (EQA) programs. Throughout the study period, all laboratories consistently performed the necessary quality control procedures according to national guidelines and international best practices. T

Regarding combination of data from different centers using the same autoanalyzer after the outlier removal, did you perform statistical analysis using the Harris and Boyd method to decide if you could combine data for the estimation of the RIs?

**Response:** No, we did not perform the Harris and Boyd method analysis. We considered this approach inappropriate due to significant differences between the individual systems and the fact that manufacturers recommend different reference intervals for their instruments. However, this method can certainly be performed if requested.

Among the limitations of the study, it has not been mentioned whether the population is iodine sufficient or not, and whether it was taken into account as a variable in the analysis.

**Response:** Our study did not include direct iodine measurements in the patient population and iodine measurements were not included as a variable in the analysis. Although iodine supplementation is provided through salt fortification and dietary sources in our country, iodine deficiency still exists in the population. (Current iodine status in Turkey. J Endocrinol Invest. 2009 Jul;32(7):617-22. doi: 10.1007/BF03346519. Epub 2009 Jun 24. PMID: 19564718.). We acknowledge this important limitation. This limitation has been added to the limitations section of the manuscript.

Were pre-analytical factors between centers standardized and assessed? Such as the time of collection, time to centrifugation, time to testing, hemolysis, icterus and lipemia indices. If it was not standardized, it should be included as a limitation of the study.

**Response:** All participating centers regularly perform assessments according to national regulations and apply standardized procedures for pre-analytical phase management. These procedures include standardized protocols for sample collection timing, centrifugation procedures, and time to testing. All participating centers are university hospitals with similar infrastructure and quality standards, which ensures consistency in pre-analytical handling across sites.

I would also suggest adding in the discussion section a comparison of the results to other publications that have previously defined thyroid hormone RI with refineR, as mentioned previously.

**Response:** The relevant publications defining thyroid hormone reference intervals using the refineR method have been comprehensively added to the discussion section. Our results have been systematically compared with previously published thyroid hormone reference intervals derived using the refineR methodology, highlighting both the similarities and differences in our findings.
