# Supplementary material for: Comparison of thyroid hormones reference intervals based on thyroid antibody levels: A multicenter study
Source: PLoS One. 2026 Mar 18;21(3):e0344197. doi: 10.1371/journal.pone.0344197 (PMC12998823; doi:10.1371/journal.pone.0344197)
Supplement: Attachment — Submitted filename: Response to Reviewers 13.02.26.docx [file pone.0344197.s005.docx]

**Response to Reviewers**

1. If the authors have adequately addressed your comments raised in a previous round of review and you feel that this manuscript is now acceptable for publication, you may indicate that here to bypass the “Comments to the Author” section, enter your conflict of interest statement in the “Confidential to Editor” section, and submit your "Accept" recommendation.

Reviewer #1: All comments have been addressed

2. Is the manuscript technically sound, and do the data support the conclusions?

The manuscript must describe a technically sound piece of scientific research with data that supports the conclusions. Experiments must have been conducted rigorously, with appropriate controls, replication, and sample sizes. The conclusions must be drawn appropriately based on the data presented.

Reviewer #1: Yes

3. Has the statistical analysis been performed appropriately and rigorously?

Reviewer #1: Yes

4. Have the authors made all data underlying the findings in their manuscript fully available?

The [PLOS Data policy](http://www.plosone.org/static/policies.action#sharing) requires authors to make all data underlying the findings described in their manuscript fully available without restriction, with rare exception (please refer to the Data Availability Statement in the manuscript PDF file). The data should be provided as part of the manuscript or its supporting information, or deposited to a public repository. For example, in addition to summary statistics, the data points behind means, medians and variance measures should be available. If there are restrictions on publicly sharing data—e.g. participant privacy or use of data from a third party—those must be specified.

Reviewer #1: Yes

5. Is the manuscript presented in an intelligible fashion and written in standard English?

PLOS ONE does not copyedit accepted manuscripts, so the language in submitted articles must be clear, correct, and unambiguous. Any typographical or grammatical errors should be corrected at revision, so please note any specific errors here.

Reviewer #1: Yes

6. Review Comments to the Author

Please use the space provided to explain your answers to the questions above. You may also include additional comments for the author, including concerns about dual publication, research ethics, or publication ethics. (Please upload your review as an attachment if it exceeds 20,000 characters)

Reviewer #1: Firstly, I would like to thank the authors for all the clarifications and adjustments they have made to the manuscript. I only have two minor comments.

Please review these sentences, as they appear to be duplicates:

L152: Moreover, reference intervals for thyroid function tests may vary not only based on antibody 153 status but also due to demographic factors such as ethnicity, age, sex, body mass index, iodine 154 status, and measurement methodology [12-14].

L161: Additionally, RI for thyroid function tests can be affected by 162 demographic characteristics (ethnicity, age, and sex), body mass index, specific medication use, 163 iodine status, and methodological differences

**Response:** We have addressed the redundancy by consolidating the information from both sentences into a single, more comprehensive statement. Specifically, we merged the details regarding antibody status, demographic factors, medication use, and methodological differences into one sentence to improve the flow of the paragraph. The redundant sentence at L161 has been removed to improve the clarity and conciseness of the manuscript.

There is a typo in:

L740: FinallyThirdly

**Response:** The typographical error has been corrected. In the revised manuscript (L746), the term "FinallyThirdly" has been replaced with "Thirdly," to match the preceding "Firstly" and "Secondly" points in the Limitations section.

7. PLOS authors have the option to publish the peer review history of their article ([what does this mean?](https://journals.plos.org/plosone/s/editorial-and-peer-review-process#loc-peer-review-history)). If published, this will include your full peer review and any attached files.

If you choose “no”, your identity will remain anonymous but your review may still be made public.


**Do you want your identity to be public for this peer review?** For information about this choice, including consent withdrawal, please see our [Privacy Policy](https://www.plos.org/privacy-policy).

Reviewer #1: **Yes:** Alicia Madurga
